# A Blockchain-Enabled Incentive Trust Management with Threshold Ring Signature Scheme for Traffic Event Validation in VANETs

**DOI:** 10.3390/s22176715

**Published:** 2022-09-05

**Authors:** Waheeb Ahmed, Wu Di, Daniel Mukathe

**Affiliations:** School of Computer Science and Technology, Dalian University of Technology, Dalian 116024, China

**Keywords:** vehicular ad hoc networks, trust, privacy, security, event validation, incentives, blockchain, ring signature

## Abstract

As a part of the intelligent transportation system, vehicular ad hoc networks (VANETs) provide timely information about road events and traffic to improve road safety and traffic efficiency. However, VANETs face many challenges, such as attacks from malicious vehicles, identity privacy leakage, and the absence of trust between vehicular nodes. In addition, vehicles nearby an event usually lack the motivation to participate in the traffic event validation whenever it occurs, which requires the cooperation of vehicles on the network. To solve these problems, a blockchain-enabled incentive trust model with a privacy-preserving threshold ring signature scheme for VANETs is proposed. Firstly, a threshold ring signature scheme is designed in order to allow participants in the non-trusted environment to anonymously witness the message’s authenticity and reliability while guaranteeing the vehicle’s privacy. Second, a blockchain-enabled incentive trust management model is presented to enable the roadside units (RSUs) to thwart various attacks and guarantee the trustworthiness of event messages transmitted in VANETs and also motivate the senders of the traffic information and their witnesses with incentives. Finally, to improve efficiency, a practical Byzantine fault-tolerant consensus mechanism is used. Our proposed system is demonstrated to be effective and secure for VANETs, according to both security analysis and performance evaluation.

## 1. Introduction

Currently, VANETs help in reducing traffic accidents, reducing traffic congestion, improving road safety, and providing a better driving experience. Safety messages and traffic information are transmitted by vehicles to other vehicles and roadside units (RSU) in order to increase drivers’ awareness [1,2,3]. However, security concerns exist in VANETs due to the fact that information is transmitted via an open network environment. This makes VANETs vulnerable to numerous types of attacks, both internal and external [4]. It is possible for malicious nodes to join the network at any time and spread false messages wirelessly in such an environment. Moreover, vehicular networks are characterized by the continuous, fast movement of vehicles, which results in the constantly changing topology of the network. Therefore, it is difficult to determine the trustworthiness of all other vehicles with which RSU interacts in a timely manner. Furthermore, vehicular networks generate a great deal of data due to their mobility and dynamic nature. Malicious vehicles can reduce the trustworthiness of these data, weakening the trust evaluation system that relies on them. Therefore, establishing a trust system for vehicles poses many challenges. First, there is a possibility that a malicious vehicle may forge traffic data to deceive others, leading to injuries and even death. Therefore, the authenticity and reliability of sent messages should be guaranteed. Second, messages may reveal personal information about participants, such as vehicle identity and position, and malicious vehicles can analyze and monitor the network messages to track other vehicles. Thus, these messages should be transmitted anonymously via the network in ideal scenarios. However, an anonymous message cannot be guaranteed to be reliable. Therefore, the ability to trace malicious vehicles should be ensured when bogus messages are detected in the network. Third, participants/vehicles can be uninterested in responding to a traffic event-validation request initiated by a sender vehicle to inform the roadside unit (RSU) when an event happens in the network. Users will lose interest in participating if there are no tangible benefits from responding to the event-validation request. Furthermore, VANETs are more prone to other malicious attacks, such as on-off attacks or collusion attacks, due to their open wireless channel characteristics and dependence on communication technologies. Therefore, there should be an effective security mechanism to address all of these issues efficiently.

As one of the most promising applications in VANETs, the threshold ring signature technique allows vehicles to detect road conditions and communicate that information (e.g., traffic jams, accidents, road constructions) to the nearest RSU for alerting other vehicles within its communication range, allowing them to avoid troublesome spots in advance. Suppose a driver is on the main road and she sees an accident on the road and wants to notify the nearest RSU so that it can alert all vehicles coming on the way to avoid the troublesome spot until it is resolved. To convince the RSU about the event, the driver needs the aid of other witnesses to issue this message together. By using this technique, the RSU can receive reliable traffic information, enabling it to broadcast traffic event notifications. The use of this technique ensures a much safer driving experience. Additionally, it reduces the frequency of accidents and traffic jams, thus reducing the expenditure of many public resources. Consequently, the information reliability of event messages plays an important role in this application. The receiver will be more willing to believe the truthfulness of an event message if there are multiple vehicles sending the same message. In addition to reducing the forwarding of duplicate messages and the waiting time for message recipients, message aggregation is an efficient way to implement majority-based authentication. Furthermore, threshold ring signature-based authentication has been found to be effective and reliable to protect the privacy of aggregate messages.

Trust management systems are introduced to enhance the security of VANETs by enabling the dissemination of reliable and trusted data in the network. They are divided into two categories: centralized and decentralized. Centralized trust management systems [5,6] use evaluation servers to analyze and store all data, which is not practical in VANETs since the majority of applications run in almost real-time and data must be delivered in a timely manner. Decentralized systems [7,8] rely on receiver vehicles and/or RSUs that evaluate sender vehicles, giving them a rating and carrying out evaluations locally. These systems reduce the overhead associated with interacting with infrastructure. Even though VANETs enable high mobility, the limited communication time between vehicles limits information exchange, and vehicles in VANETs are usually strangers and cannot be fully trusted. In addition, existing trust management models suffer from the effect of collusion and on-off attacks performed by malicious vehicles. Therefore, ensuring the trust of transmitted event messages in VANETs is an issue that needs to be addressed efficiently.

To incentivize vehicles to exchange correct data, VANETs require incentives. Vehicles can be malicious and refuse to participate and respond to the request of validating the event without any motivation. It is necessary to develop incentive mechanisms to encourage vehicles to cooperate and validate events. Misbehaving vehicles may be punished via a reduction in their trust score and/or revocation after they have reached a certain threshold of misbehavior. Furthermore, the storage of trust values should be addressed efficiently. Traditional storage systems are centralized and vulnerable to single points of failure. On the other hand, blockchain technology is a distributed ledger [9] that prevents data from being altered and allows data to be stored in a distributed manner. The huge number of vehicles, the strict requirements about delays, and the restricted geographical region in which specific vehicular information is found have all resulted in the high interest in distributed trust management systems.

Blockchain is emerging as a promising solution for managing trust evaluation in vehicular networks due to its key characteristics, which include decentralization, tamper-proof, consistency, and fault tolerance. Blockchain-based trust management systems provide better performance evaluations, improved capabilities, and information transparency to withstand malicious attacks [9,10].

Motivated by the issues mentioned above, in this paper, we propose a blockchain-based incentive trust management system in combination with a threshold ring signature scheme as a means of establishing secure vehicular communication.

In summary, the main contributions of this paper are summarized as the following:

- First, a threshold ring signature scheme is designed for practical use in VANETs; it utilizes an identity-based ring signature to maintain privacy, message authenticity, and reliability, and achieve efficiency.
- Second, we developed a blockchain-enabled incentive trust management model that ensures trust in the communications of VANETs and improves the security of the network. The system is able to aggregate and propagate trust values to improve scalability, thwart security attacks, and trace and revoke malicious vehicles. In addition, the consensus mechanism is developed to efficiently reduce the costs associated with the traditional public blockchain.
- To incentivize vehicles to participate in the event-validation request, we proposed an incentive mechanism. A reward is given to the sender vehicles and their witnesses. Vehicles that agree to the request are only given incentives. It encourages vehicles to cooperate and send correct traffic information and discourages them from sending false information and acting maliciously.
- We conducted the security analysis and performance evaluation of our system to demonstrate that it is secure, robust, and efficient for vehicular networks.

The rest of the paper is structured as follows: Section 2 discusses the related works. Section 3 introduces the proposed system model. The proposed system details are presented in Section 4. Section 5 elaborates the security analysis. The performance evaluation is discussed in Section 6. Section 7 presents the discussion. Finally, Section 8 concludes the paper.

## 2. Related Works

*A. Authentication and Blockchain-Based Schemes for VANETs*

The open environment of VANET makes it vulnerable to a variety of security, privacy, and trust attacks. Trust management is essential to identifying malicious vehicles, determining the reliability of traffic data, and disseminating trustworthy messages in the network. Yang et al. [11] proposed a blockchain-based framework that addresses the problem of fake events and vehicle trustworthiness by obtaining signatures from adjacent passing vehicles and verifying the event. The proposed mechanism can detect malicious nodes and prevent the spreading of fake events throughout the network. This system uses reputation value to detect false information and proof-of-event to achieve consensus.

Blockchain-based schemes are proposed in [12,13,14] to maintain peer trust and securely shared data. These techniques, however, have high computational and storage requirements. The above schemes, however, are vulnerable to collusion attacks and on-off attacks in which a malicious vehicle with a high reputation distributes false information, then switches its behavior and distributes true information to boost its trust score again.

Khelifi et al. [15] developed a reputation-based blockchain solution for the purpose of securing information and data forwarding and caching. In order to maintain the reputation of the authentic vehicles, a lookup table is maintained. The problem with this approach is that it is vulnerable to on-off attacks and collusion attacks.

Malik et al. [16] introduced a new VANET trust management system that compares the received signal strength indicator, the packet delivery rate, and the renewable partnership for a vehicle to a threshold value in order to establish the vehicle’s credibility. As trust values are openly available, this system is vulnerable to tracking and privacy leaks.

Roy and Madria [17] proposed a misbehavior-detection and event-validation framework based on blockchain for detecting malicious vehicles and valid traffic events based on neighboring vehicles’ information and the events reported by individual vehicles. However, it cannot deal with on-off attacks.

Ayobi et al. [18] propose a decentralized trust mechanism based on blockchain for maintaining anonymity in VANET. Messages received by vehicles are evaluated based on the reputation score of the sender and the distance from the sender to the event location. It can determine whether the messages received are valid by applying the Dempster-Shafer theory. For each message they receive from the sender vehicle, nodes create trust values, and RSUs combine the trust values to produce accurately reported events. RSUs will eventually store trustworthy messages in the cloud and append the hash of the data to the blockchain. However, it is not able to cope with on-off attacks.

To improve the safety of VANETs, Yan et al. [19] proposed a trust model based on statistical methods. Using statistical concepts such as significance tests, hypothesis tests, and confidence intervals, the model assists both the reputation management center and the vehicle nodes in calculating trust values for all vehicle nodes and determining whether or not a message can be trusted. However, this trust model is only capable of detecting false message attacks.

Awan et al. [20] introduced a trust management-based secure energy sharing mechanism that computes trust degrees. A multi-leveled centralized system is proposed that uses infrastructure as well as vehicles to maintain a safe environment. To improve scalability, the suggested vTrust aggregates and propagates the degree of trust. The node requesting energy resources is required to maintain a certain level of trust in order to earn resources. This scheme identifies malicious vehicles in the network, but it does not satisfy the privacy requirement of VANET.

Jiang et al. [21] used a sliding time window algorithm to measure the degree of vehicle trust based on the trust values of the vehicles at various time intervals, and they used a penalty factor to guard against sudden attacks from malicious nodes. A DQN-based path prediction algorithm is also proposed to facilitate trust-sharing, allowing RSUs to share the relevant vehicle’s trust information. This approach is designed to thwart false information attacks.

Wang et al. [22] presented a trust-based and secure method for sharing real-time traffic information. The RSU requests real-time traffic data from nearby vehicles. Upon receiving the road information from the reporting vehicle, the RSU verifies the legitimacy of the vehicles, and it will then send all of the vehicles’ messages to the TA by signatures aggregation. The TA calculates the trust values of the vehicles to prevent malicious messages from spreading. This approach is designed to thwart Sybil attacks and false information attacks. However, it requires centralized trust computation, which is not suitable for the distributed nature of VANETs.

The above schemes do not take into account the incentives to motivate vehicles to share traffic information and to prevent their selfishness. Additionally, revocation is not addressed against possible malicious vehicles performing different malicious attacks.

In order to achieve the information interaction security of VANETs, Wang et al. [23] presented a blockchain-assisted trustworthiness calculation of vehicles to establish vehicle-to-infrastructure (V2I) authentication that took the handover scenario into account in a decentralized manner. They adopted bilinear pairing operations, which have a high computational cost and their scheme is vulnerable to numerous security attacks launched by malicious vehicles.

Lin et al. [24] designed a new system consisting of signatures of knowledge (SoK), key derivation (KeyDer), smart contract, and a blockchain-based authentication scheme. This scheme satisfies many of the security requirements of VANETs. However, it has a high computational cost.

In [25], a group signature protocol based on lattice cryptography is proposed for authentication in VANETs. The authors use quantum-resistant and Bonsai-tree signatures to achieve forward security in VANETs.

The authors in [26] introduce a self-blindable signature-based end-to-end anonymous key exchange system. For each transmission beyond the mix-zone, vehicles in this protocol first secretly blind their own private certificates, after which they compute an anonymously shared key based on proof of knowledge (PoK). Their protocol accomplishes secure authentication.

In [27], a new, conditional, privacy-preserving, certificateless aggregate signature scheme for VANETs is proposed that uses full aggregation technology. This scheme can ensure the authenticity of transmitted information, protect vehicle privacy, save on computation and bandwidth resources and resist some security attacks.

The previously mentioned schemes do not satisfy the trust requirement of VANETs as they are vulnerable to false message attacks.

Yu et al. [28] proposed a new intrusion-detection system (IDS) based on deep learning and time series classification. From events messages reported by vehicles near traffic incidents, they gather time-series feature vectors of traffic parameters that are directly related to traffic events. Using time-series feature vectors from both collusion and normal attack scenarios, a long short-term memory (LSTM)-based traffic event classifier is constructed and trained to more precisely determine how traffic parameters have changed over time. This scheme can detect false message attacks. However, it does protect the privacy of vehicles, but cannot trace and revoke malicious vehicles.

He et al. [29] study and examine the issue of delay-sensitive secure transmission in the unmanned aerial vehicle (UAV)-relayed VANETs. They take into account the security assurance and describe this issue as a total information delay minimization problem by optimizing the channel allocation and UAV relay trajectory. The channel allocation is determined via relax-and-round and sequential convex approximation methods, and the UAV relay trajectory is solved using the Newton method.

For mobile edge computing (MEC)-enabled UAV-assisted VANETs, He et al. [30] formulate a multi-objective optimization problem that simultaneously considers task offloading, resource allocation, and security assurance to minimize the task processing delay. The multi-objective optimization problem has been decoupled into two subproblems. Their iterative algorithm effectively solves the joint optimization problem by combining the relax-and-rounding and Lagrangian methods.

*B. Synthesis*

VANETs’ safety-related applications impose restrictions in terms of real-time processing. Processing delay on each vehicle or RSU must be maintained to an absolute minimum due to the time-sensitive nature of the information. Additionally, vehicles encounter intermittent connectivity because of their high mobility. This suggests that a message must be created rapidly enough to be sent before the short communication ends.

However, a high traffic density environment leads to a large number of vehicles (100–200) moving at high speed within the RSU’s communication range. Thus, the associated RSU will need to verify more signatures every 100–300 ms. As a result, the RSU is subjected to considerable processing load, leading to delays in the authentication of messages, resulting in performance degradation. There can also be a loss of connectivity between the vehicles and the RSU. Additionally, if malicious vehicles/adversaries launch various attacks on the network (collusion, on-off attacks, and other attacks), the authenticity and reliability of messages sent by vehicles to the RSU can also affect network security. Furthermore, compromised RSUs may alter, delete or cause damage to the data stored on them.

A few drawbacks and challenges still remain for existing anonymous authentication schemes as most schemes use expensive cryptographic operations such as map-to-point hash functions and bilinear pairing operations, which leads to an increase in computational costs and delays. When authenticating traffic-related messages in VANETs, these operations take much time. To increase efficiency in VANET environments, schemes must be developed without using bilinear pairing operations. Thus, the design of a ring signature scheme based on Lagrange interpolation that can efficiently handle high traffic density environments and has less computational costs has recently become a hot topic in VANETs.

Furthermore, none of the existing authentication schemes described in the literature (e.g., [31,32,33,34,35]) satisfy the necessary levels of security, privacy, trust, and computational efficiency simultaneously.

Moreover, we have noticed that most of the existing schemes on trust management in VANETs have not addressed the protection of the privacy of the vehicles (anonymity) since vehicles’ real identities can easily be leaked when the vehicles interact with one another or with the RSU and are unable to resist collusion (a large number of vehicles launch attacks together) and on-off attacks or trace the real identities of malicious vehicles and revoke them from the vehicular network, so the previous trust management protocols have become somewhat impractical.

Furthermore, existing trust management models require efficient blockchain-based incentives and reward mechanisms to motivate vehicles to report reliable traffic data to the RSU and prevent them from acting maliciously.

Current decentralized trust management schemes suffer from untimely synchronization of trust data between RSUs and the costs associated with the traditional public blockchain. Therefore, a consensus method with better performance is required.

To fill the security, privacy, and trust gaps in VANET, an effective solution that combines a threshold ring signature scheme with a blockchain-enabled incentive trust model is proposed to address the drawbacks of traditional authentication schemes and trust models.

The threshold ring signature scheme is created to offer flexible anonymous authentication, allowing it to successfully achieve reliability, privacy, authentication, and computational efficiency at the same time. Furthermore, the blockchain-enabled incentive trust model aims to achieve privacy, trustworthiness, traceability, and revocation. Moreover, it can resist collusion and on-off attacks and can protect the identities of legitimate vehicles and track the identities of malicious vehicles, thereby enhancing security. It also increases the users’ enthusiasm by providing incentives to vehicles to actively participate in traffic events validation. The blockchain is adopted due to additional features such as decentralization, robustness, tamper resistance, flexibility, immutability, transparency, anonymity, etc. To address the issue of untimely trust values synchronization in VANET and the cost of block creation, we developed a consensus mechanism based on a practical Byzantine fault-tolerance (PBFT) algorithm for achieving better efficiency.

## 3. System Model

**Figure 1:**
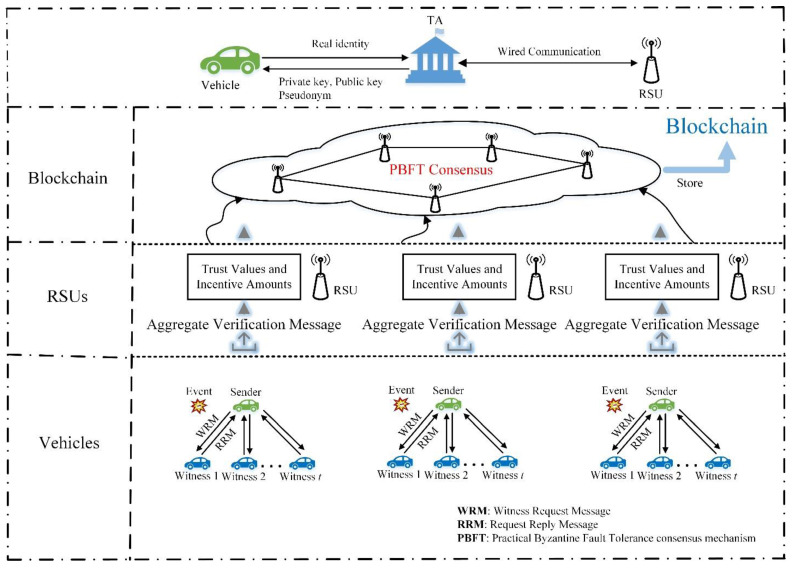
System architecture.

This section presents the system architecture. Figure 1 depicts the system architecture, which includes the trusted authority (TA), vehicles (the sender (S) and witness (W)), receivers (RSUs), and blockchain. Then, we discuss the adversary model and design goals in detail.

*A. System Architecture*

*Trusted Authority:* The TA generates each vehicle’s private key, public key, and pseudonym, and maintains a database of vehicles’ real identities, public keys, and pseudonyms to trace it in case of malicious activity. To participate in the VANET, legitimate vehicles and RSUs must be authorized by TA, which is a fully trusted entity. It is also in charge of tracking vehicles in case of malicious activity.

*Vehicles:* Vehicles are equipped with an On-Board Unit (OBU) that enables communication within the network. In a specific incident, the vehicles can be categorized as a sender or witness. When vehicle S finds an accident, it would like to inform the RSU nearest to it. A request will be sent by S to the witnesses asking them to confirm the initiated event with their signatures. Vehicle W is driving near the corresponding event and sends a witnessing message to S. Verifying the authenticity of witness messages requires a set of witnesses (W1,⋯,Wn). The witnesses confirm the event by signing the message and responding to the sender with their signatures.

*RSUs*: RSUs are roadside devices that facilitate communication between vehicles, TA, and other RSUs. These RSUs are assumed to have sufficient storage and computing capabilities. RSUs are the miners in the blockchain. They issue transactions and store a copy of the full blockchain. After RSU receives traffic event reports submitted by sender vehicles, it needs to calculate the trust values of the sender vehicles and evaluate the traffic event reports/aggregate messages’ authenticity, reliability, and trustworthiness, and rewards monetary incentives to the participating vehicles and their witnesses if the reports are credible. Then, RSU broadcasts an event notification to the vehicles within its communication range to take action. Due to their vulnerability to crash or compromise, RSUs are considered semi-trusted.

*Blockchain*: With practical Byzantine fault-tolerant (PBFT) consensus and identically distributed copies of the ledger, blockchain is extremely secure and reliable. Our VANET design uses blockchain to improve security.

The proposed system consists of two components and aims to build privacy-preserving threshold authentication and a trust management scheme that guarantees the authenticity, reliability, and trustworthiness of information disseminated in VANET. The first component of the proposed system is a threshold ring signature scheme. In a VANET environment where the vehicles are not fully trusted, this scheme ensures the authenticity, reliability, and privacy of aggregate messages. The aggregation of messages in VANETs is an effective solution for achieving threshold authentication and reducing network overhead. The second component of this system is the blockchain-enabled incentive trust model. When an event occurs, the sender (vehicle) sends out a message announcing an event and inviting other vehicles to agree as witnesses. If they accept the sender’s signature, witnesses respond with corresponding signatures. The sender creates an aggregation message (in order to achieve anonymity) with witness-signed replies and sends the aggregate message to the nearest RSU for verification. Multiple senders will send event messages to the nearest RSU. The RSU calculates the trust values of each sender. In order to determine whether the reported aggregate messages are true or not, the trust value is used. Senders with a trust value greater than the threshold are rewarded along with their witnesses with incentives to encourage users to share traffic information. Then, an RSU is selected as a primary node to add the block of trust values to the blockchain by utilizing PBFT to verify the validity of the block by collaborating with authorized RSUs.

*B. Adversary Model*

Despite the fact that TA has registered both vehicles and RSUs, compromised RSUs and malicious vehicles may exist. Our discussion will be focused on two types of adversaries:

(A) Malicious vehicles: Malicious vehicles are vehicles that have the capability to communicate and carry out several kinds of attacks, including:
  (1) False messages attack: To mislead or disturb their neighbors, malicious vehicles send out false event messages. A selfish vehicle, for example, may claim the road is blocked when it is not, and then suggest that others divert so the route can be cleared of traffic so the vehicle can drive fast. Another example is a malicious sender and a witness who wants to acquire the monetary incentive from RSU without having to provide real traffic data.
  (2) Collusion attack: Several vehicles coordinate their attacks in VANET to confirm a false event message by launching attacks together [36].
  (3) On-off attack: Malicious vehicles display multiple modes of behavior: they engage in dishonest behavior (where they initiate an attack) for some time, then they engage in honest behavior (where they establish a higher trust score) to avoid detection. Intelligent attackers use on-off attacks to cause damage while avoiding being identified and ejected from vehicular networks [37]. To prevent such attacks from occurring, our proposed scheme uses adaptive-detection thresholds to detect bad behavior.
  (4) Sybil attack: The malicious vehicle/adversary uses multiple pseudonyms to generate numerous feedbacks on a single event leading the receivers to believe that the messages are coming from various sources and that the content of the messages is accurate in order to affect trust assessment.
(B) Compromised RSUs: The RSUs are usually located along roads and are vulnerable to physical attacks. Therefore, RSUs are assumed to be semi-trusted, and they might be exploited by attackers to change or delete data and offer services to anyone. If the attacker has control of the RSU, it may refuse to mine blocks, reducing the system’s robustness and efficiency. Several hacked RSUs work together to insert a bogus data block into the blockchain. Due to the high cost, we assume an attacker will be able to compromise only a small proportion of the RSUs.

*C. Design Goals*

Our design goals mainly include the following:

(1) Decentralization: The incentive, trust score computation, and updating are done by RSUs in a decentralized environment without the involvement of a third party (trusted or otherwise). The proposed system should have no single point of failure.
(2) Enthusiasm: Our system offers incentives to senders and witnesses in order to motivate them to cooperate with the event-validation request. If the sender and witnesses are honest, the RSU will acquire the real traffic information, and senders and witnesses earn the incentives as a reward.
(3) Reliability and Robustness: An adversary cannot alter or forge the message of the sender and witness. The content of the event messages sent by senders and the trust data stored in the RSUs are unlikely to be tampered with (tamper-resistance). An attacker should not be able to deceive the trustworthiness assessment or disable its functionality. The authenticity and trustworthiness of an alert message should be guaranteed.
(4) Privacy-preserving: No information about the identity of the sender or witnesses should be revealed in the request or the response.
(5) Processing Time: It is essential that the proposed system should have a processing and execution speed of a few milliseconds, ensuring that transactions are processed and that the updated trust ratings are provided to the system in a timely manner.
(6) Traceability and Revocation: Vehicles that abuse the VANET should be traced by the TA. In addition, when a misbehaving vehicle is identified, the TA should be able to revoke it immediately. In this way, the misbehaving/malicious vehicle cannot cause further damage.
(7) Efficiency: To make VANETs economically viable, OBUs have resource-limited processors. Thus, the cryptography operations used during authentication should incur only a minor computational cost.

## 4. System Details

The proposed system consists of two components: the TRS scheme and the blockchain-enabled incentive trust model. The system architecture is shown in Figure 1.

*A. Threshold Ring Signature Scheme*

The threshold ring signature (TRS) scheme requires at least t out of n ring members to jointly generate a signature, without leaking their real identities. In other words, for a message to be valid in a (t,n)- TRS scheme, a minimum of t out of n members should attest and sign the message, while the actual signer remains anonymous. A typical TRS scheme is made up of two steps.

TRS.Sign( ): Takes as input a message m, threshold value t, number of ring members n, public keys for n members, and private keys for t members, and generates a (t,n)- TRS signature σ on message m.

TRS.Verify( ): Takes as input message *m*, threshold ring signature *σ*, and public keys for *n* members, and outputs 1 if the (*t*,*n*)- TRS signature *σ* on message *m* is valid, and 0 otherwise.

A typical TRS scheme is fully deterministic in the sense that the ring members are pre-selected, trust each other, and can share private keys. However, vehicles in VANETs are non-trusted users and can join and leave the network dynamically. Thus, the typical TRS scheme is not practical in the VANET environment.

The Proposed TRS Scheme

The proposed TRS scheme is designed to suit VANET (i.e., a non-trusted, non-deterministic, and dynamic environment).

Communication Scenario

Suppose vehicle $V_{A}$ iss an eyewitness of an accident and would like to send an event message to the nearest RSU so the RSU can alert other vehicles in its communication range. To guarantee message trustworthiness, vehicle $V_{A}$ needs witnesses to attest to the event’s occurrence. The notion is that the more the number of witnesses, the more the receiver RSU of the message believes it. First, vehicle $V_{A}$ requests the nearby vehicles to become witnesses in his event message and sets a threshold value of t. When t−1 vehicles reply with messages attesting the event message is true, the vehicle $V_{A}$ composes an aggregate message with t attestations and sends it to the nearest RSU. The RSU will verify the reliability and trustworthiness of this aggregate message. Then, it will broadcast an event notification to the vehicles in its communication range. Suppose vehicle $V_{B}$ is an incoming vehicle and receives the event notification, she drives with caution as she approaches the accident scene, or she can consider changing her entire route.

**Figure 2:**
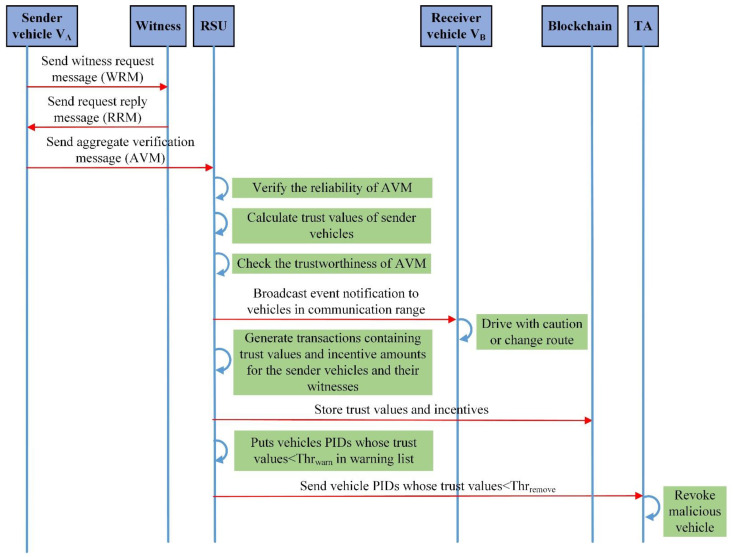
Validating the reliability and trustworthiness of reported road incidents.

The UML sequence diagram in Figure 2 shows the flow of events in the communication scenario.

Types of Participants and Messages

There are four types of participants in the proposed TRS scheme: a trusted authority TA who generates each vehicle’s private key, public key, and pseudonym, a sender S who detects and broadcasts event messages, a witness W who confirms the occurrence of an event message, and a receiver R (i.e., RSU) who verifies the event message. Depending on the type of participant, three types of messages are generated.

1. Witness-Request Message (WRM): upon detecting a traffic-related event, the sender S broadcasts a four-tuple WRM (traffic-related message msg, threshold value t, ring-size value r, cryptographic content π) to potential witnesses. Multiple vehicles may send WRM, but only a few will receive replies, i.e., request-reply message (RRM). We refer to the vehicle which receives RRMs as the sender S.
2. Request-Reply Message (RRM): after receiving the WRM from the sender S and verifying it, a vehicle becomes a witness W by sending an RRM to the sender S. The RRM contains the signature and identity of the witness *W*. A witness may simultaneously receive multiple WRMs about the same traffic-related event from multiple senders.
3. Aggregate-Verify Message (AVM): After receiving t−1 RRMs, the sender S composes an AVM and sends it to the receiver (i.e., the nearest RSU), who verifies and take action per the traffic-event message.

Framework

**Figure 3:**
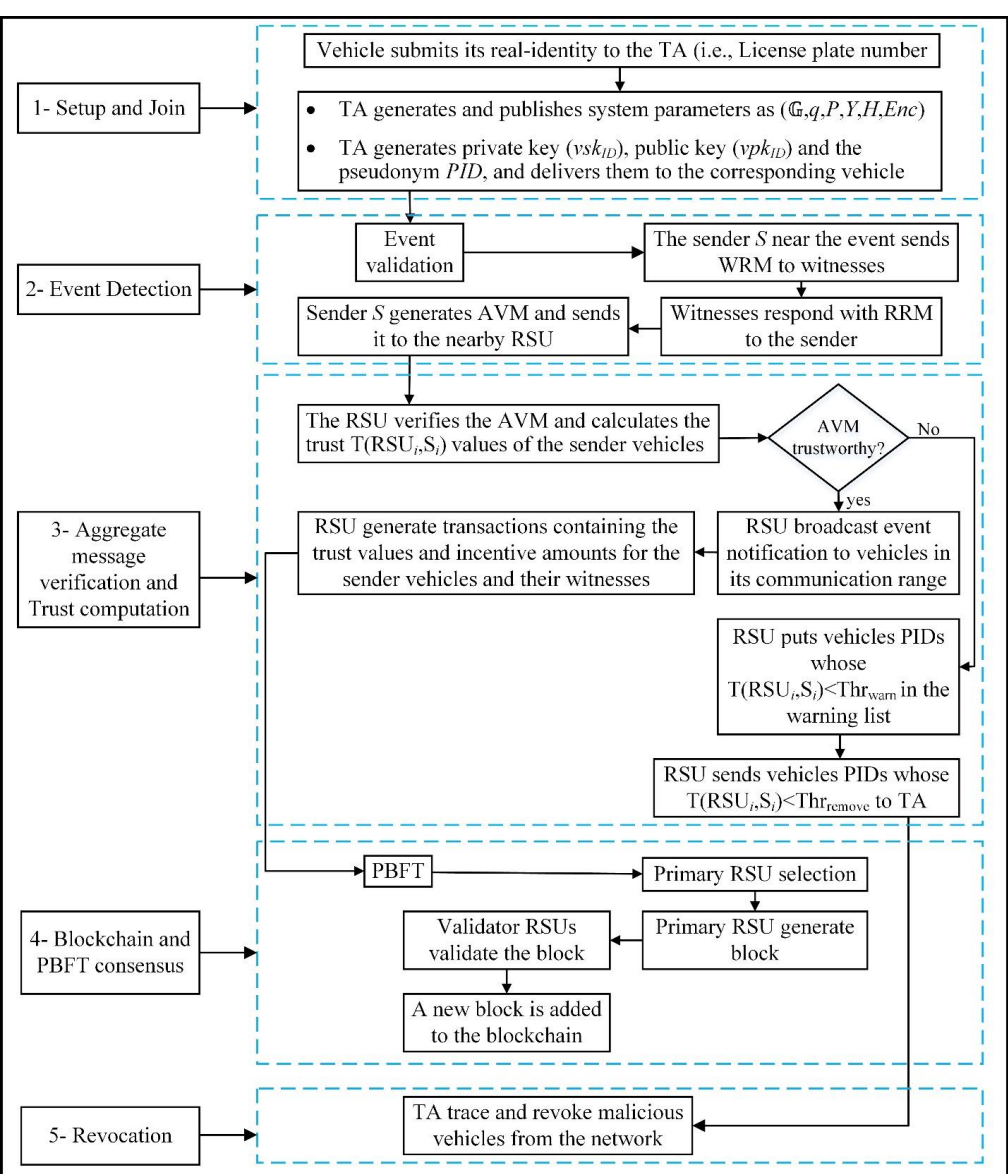
The framework of the proposed system.

The proposed TRS scheme comprises seven phases: setup, join, event detection, witness request, request-reply, aggregate message generation, and aggregate message verification. The TA executes the setup phase to generate public system parameters and cryptographic keys for the vehicles. It preloads them into the vehicle’s TPD during the join phase. The sender S is responsible for the event-detection and witness request phases. The sender S detects a traffic-related event and composes an event message during the event-detection phase. In the witness request phase, sender S chooses some system parameters and broadcasts WRMs to other vehicles calling them for witnesses. The witness runs the request-reply phase by replying to the sender S with RRMs, ideally a fraction of a ring signature. After receiving t RRMs, the sender S enters the aggregate message generation phase, where it sends an AVM to the nearest RSU. The last phase is the aggregate message verification, where any receiver RSU can verify the AVM as shown in steps 1, 2, and 3 in Figure 3. Next, the RSU performs the trustworthiness check on the received AVM using the blockchain-incentive trust model as depicted by steps 3, 4 and 5 in Figure 3.

Design

We recommend the architectural views model in [38] to describe the software architecture and system integration. The model encompasses various architectural views, including integrated processes, logical, deployment, use cases, contracts, and integrated services. We focus on logical and deployment views to help comprehend the information exchange between systems. The logical view realizes the functions of the software system specified in the use cases. Typically, three UML diagrams can be used for this purpose: the class diagram, the sequence diagram, and the communication. On the other hand, the deployment view is associated with the arrangement of software components into hardware components and the physical runtime installation of the software system. The software installation requires both hardware and execution environments to operate. We can use a UML deployment diagram. A stereotype is a new type of modeling element that extends the semantics of existing elements in the UML metamodel. Stereotypes have been applied to nodes, components, and communication protocols.

**Figure 4:**
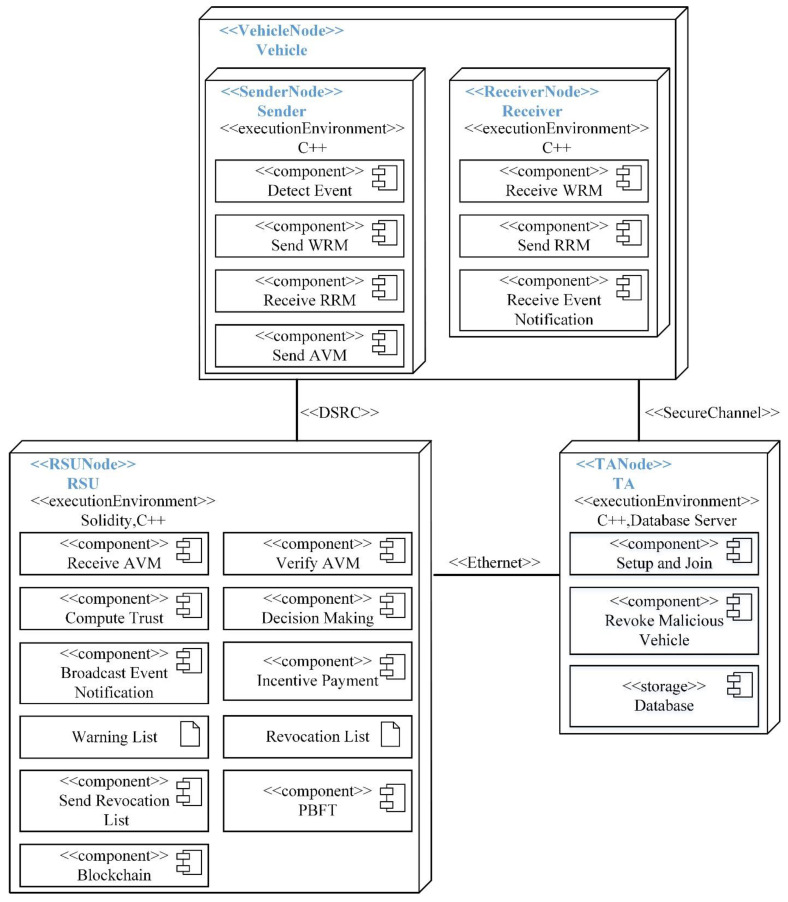
The UML deployment diagram of the proposed system.

In the proposed solution, nodes (i.e., vehicles, RSU, and TA) are labeled with appropriate stereotypes. For instance, the vehicle node is labeled ≪VehicleNode≫, the RSU nodes are labeled with ≪RSUNode≫, and the TA node is labeled with the ≪TANode≫ as shown by the UML deployment diagram in Figure 4. Typically, nodes host and execute the components. The vehicle node in our scheme serves multiple purposes, including sender and receiver. When a node acts as a sender, it is responsible for detecting traffic events, creating and sending WRM and AVM messages, or receiving RRM messages. On the contrary, when acting as a receiver, it generates and transmits RRM messages or receives event notifications. The vehicle node can interact with other nodes. Among the duties of the RSU node are receiving AVM messages, verifying them, computing trust values of sender vehicles, generating incentives, warning lists, revocation lists, broadcasting event notifications, running the PBFT consensus (generating blocks and adding them to the blockchain ledger), hosting the blockchain ledger and sending the revocation list to the TA. The TA node is mandated to register vehicles, generate system parameters, host a vehicle information database (such as public key, pseudonym and real identity), and revoke malicious vehicles. Nodes in the network communicate via protocols, which are also represented using stereotypes. These communication protocols include dedicated short-range communication (DSRC) for enabling vehicle-to-vehicle and vehicle-to-RSU communications.

Among the duties of the RSU node are receiving AVM messages, verifying them, computing trust values of sender vehicles, generating incentives, warning lists, revocation lists, broadcasting event notifications, running the PBFT consensus (generating blocks and adding them to the blockchain ledger), hosting the blockchain ledger and sending the revocation list to the TA. The TA node is mandated to register vehicles, generate system parameters, host a vehicle information database (such as public key, pseudonym, and real identity), and revoke malicious vehicles. Nodes in the network communicate via protocols, which are also represented using stereotypes. These communication protocols include dedicated short-range communication (DSRC) for enabling vehicle-to-vehicle and vehicle-to-RSU communications. The RSU nodes and the TA node communicate via the Ethernet connection, whereas the vehicle nodes communicate with the TA node using a secure channel. 

Below are details of the proposed TRS scheme.

1. **Setup**

Let point P on an elliptic curve be a generator of a cyclic additive group 𝔾 with order q. Let c←Enc(k,m) be a symmetric encryption scheme, where k is the secret key, m is the plaintext and c is the ciphertext and $m\leftarrow Enc^{-1}\left(k,c\right)$ is a correct decryption scheme. Let H be a general one-way hash function. The TA runs the setup as follows.

- Generates n random numbers $x_{i}\in Z_{q}^{}$, and for every $x_{i}$, i=1,2,⋯,n computes $Y_{i}=x_{i}P$.
- Defines $X=\left(x_{1},x_{2},\cdots ,x_{n}\right)$ as the master private key space and $Y=\left(Y_{1},Y_{2},\cdots ,Y_{n}\right)$ as the master public key space.
- Chooses four general one-way hash functions $H_{0}:\{0,{1\}}^{}\to \{0,{1\}}^{n}$, $H_{1}:G\to Z_{q}^{}$, and $H_{2},H_{3}:\{0,{1\}}^{}\to \{0,{1\}}^{l}$, where n,l are fixed numbers of bits.
- Publishes public system parameters as (G,q,P,Y,H,Enc).

2. **Join**

The join phase is run by the TA before vehicles begin to send and receive event messages. For each vehicle with a verified identity ID, (e.g., a license plate number issued by a motor vehicle manufacturer), the TA generates the vehicle’s public, private key pair, and the pseudonym as follows.

- Computes private key $vsk_{ID}=\sum_{i=1}^{n}h_{i}x_{i}$ mod q, $H_{0}\left(ID_{i}\right)$ for i=1,2,⋯,n.
- Computes public key $vpk_{ID}=\sum_{i=1}^{n}h_{i}Y_{i}$, where $h_{i}$ is the ith bit value of a n-bit string; $H_{0}\left(ID_{i}\right)$ for i=1,2,⋯,n.
- Chooses randomly $k\in Z_{q}^{}$ and computes K=kP.
- Computes $z_{i}=x_{i}K$, and $PID=\sum_{j=1}^{n}h_{j}z_{i}$, where $h_{j}$ refers to the jth bit value of a n-bit string; $H_{0}\left(ID_{i}\right)$ for i,j=1,2,⋯,n. The PID acts as the pseudonym of the vehicle.
- Finally, the TA delivers the $vsk_{ID}$, $vpk_{ID}$ and the PID to the corresponding vehicle through a secure channel.

3. **Event Detection**

When a traffic event occurs, the sender S composes a message msg describing the incident. Meanwhile, the sender S defines a threshold value t and a ring-size r based on the number of vehicles within the communication range. The members of the ring r form an anonymous group and mix with a bigger group to provide the privacy of the actual signers.

4. **Witness Request**

In this phase, the sender S generates the witness-request message (WRM) which contains the cryptographic content π using the following steps.

- Chooses randomly r−t identities from a set of verified IDs and define set $\overset{´}{A}=\left\{ID_{1},ID_{2},\cdots ,ID_{r-t}\right\}$. For every $ID_{i}\in \overset{´}{A}$, computes new public key $VPK_{i}=\sum_{j=1}^{n}h_{j}Y_{j}$, where $h_{j}$ refers to the jth bit value of a n-bit string; $H_{0}\left(ID\right)$ for j=1,2,⋯,n.
- For every $ID_{i}\in \overset{´}{A}$, assigns a random index $r_{i}\in Z_{q}^{}$.
- For every $ID_{i}\in \overset{´}{A}$, chooses random values $a_{i},b_{i}\in Z_{q}^{}$ and create a fake identity that is indistinguishable with $VPK_{i}$ by computing $\mu_{i}=a_{i}P+b_{i}VPK_{i}$, $\vartheta_{i}=-b_{i}^{-1}H_{1}\left(\mu_{i}\right)$, and $m_{i}=a_{i}\vartheta_{i}$. The $\left(\mu_{i},\vartheta_{i}\right)$ is a valid EC-ElGamal signature of the message $m_{i}$ with verification equation $m_{i}P=$ $H_{1}\left(\mu_{i}\right)VPK_{i}+\vartheta_{i}\mu_{i}.$
- Defines cryptographic content π as given below.
  $$\pi =\left(\begin{array}{c} \left\{ID_{1},ID_{2},\cdots ,ID_{r-t}\right\},\left\{r_{1},r_{2},\cdots ,r_{r-t}\right\},\left\{\mu_{1},\mu_{2},\cdots ,\mu_{r-t}\right\}\\ \left\{m_{1},m_{2},\cdots ,m_{r-t}\right\},\left\{\vartheta_{1},\vartheta_{2},\cdots ,\vartheta_{r-t}\right\} \end{array}\right)$$
- The sender S broadcasts a tuple (msg,t,r,π) as the WRM to other vehicles calling them to be witnesses.
  $$\mathrm{Proof of Correctness}\;\begin{array}{ll} m_{i}P & =a_{i}P\vartheta_{i}\\ & =\left(\mu_{i}-b_{i}VPK_{i}\right)\vartheta_{i}\\ & =\left(\mu_{i}-b_{i}VPK_{i}\right)-b_{i}^{-1}H_{1}(\mu_{i})\\ & =\left(-b_{i}^{-1}H_{1}\left(\mu_{i}\right)\mu_{i}\right)+H_{1}\left(\mu_{i}\right)VPK_{i}\\ & =H_{1}\left(\mu_{i}\right)VPK_{i}+\vartheta_{i}\mu_{i} \end{array}$$

5. **Request-Reply**

Upon receiving the WRM tuple (msg,t,r,π) from the sender S, a witness W checks the correctness of WRM by verifying the equation $m_{i}P=$ $H_{1}\left(\mu_{i}\right)VPK_{i}+\vartheta_{i}\mu_{i}$ for each $ID_{i}$. If all the tuples $m_{i},\vartheta_{i},\mu_{i}$ satisfy the verification equation, the witness W accepts the validity of the WRM generated by the sender S. Otherwise, rejects. Next, the witness W executes the below steps.

- Computes a symmetric key $k=H_{2}\left(msg\right)$. The key size of k is l bits.
- Constructs a polynomial f over $GF\left(2^{l}\right)$ that meets conditions: deg(f)=r−t, $f\left(0\right)=H_{3}\left(t∥r\right)$, and $f\left(r_{i}\right)=Enc\left(k,m\right)$ for i=1 to r−t.
- Select random index $r\in Z_{q}^{}$. If $r\notin \left\{r_{1},r_{2},\cdots ,r_{r-t}\right\}$, compute $m=Enc^{-1}\left(k,f\left(r\right)\right)$. Else, abort the process.
- Pick a random value $c\in Z_{q}$ and compute μ∈cP and $\vartheta =\left(m-skH_{1}\left(\mu \right)\right)c^{-1}$. Sets (μ,ϑ) as the EC-ElGamal signature of m.
- Finally, the witness W sends a tuple (ID,r,m,(μ,ϑ),PID) as reply-request message (RRM) to the sender S.

6. **Aggregate Message Generation**

Upon receiving t RRMs from witnesses, the sender S generates an aggregate-verify message (AVM) as follows.

- Let set $A=\left\{ID_{r-t+1},ID_{r-t+2},\cdots ,ID_{r}\right\}$ represent identities of the witnesses.
- Mixes the signatures in the RRMs with the fake signatures in the WRM to generate a threshold ring signature on AVM as given below.
- $AVM=\left(msg,t,A\cup \overset{´}{A};\langle PID_{1},r_{1},m_{1},\mu_{1},\vartheta_{1}\rangle ,\langle PID_{2},r_{2},m_{2},\mu_{2},\vartheta_{2}\rangle ,\cdots ,\langle PID_{r},r_{r},m_{r},\mu_{r},\vartheta_{r}\rangle \right)$.
- Eventually, the sender S broadcasts the AVM to notify other vehicles of the event. Note that witnesses’ identities (i.e., signers of AVM) are indistinguishable from the bigger group. Therefore, anonymity is achieved.

7. **Aggregate Message Verification**

Any receiver RSU can verify an AVM as follows.

- Inputs $AVM=\left(msg,t,A\cup \overset{´}{A};\langle PID_{1},r_{1},m_{1},\mu_{1},\vartheta_{1}\rangle ,\langle PID_{2},r_{2},m_{2},\mu_{2},\vartheta_{2}\rangle ,\cdots ,\langle PID_{r},r_{r},m_{r},\mu_{r},\vartheta_{r}\rangle \right)$.
- For each $PID_{i}$ (i=1,2,⋯,r), the RSU verifies whether the verification equation $m_{i}P=$ $H_{1}\left(\mu_{i}\right)PID_{i}+\vartheta_{i}\mu_{i}$ holds. If any of the tuples $\langle m_{i},\mu_{i},\vartheta_{i}\rangle$ does not satisfy the verification equation, the RSU rejects the AVM. Otherwise, it continues with the verification as below.
- Computes a symmetric key $k=H_{2}\left(msg\right)$.
- For every $ID_{i}$, i = 1 to r, computes public key $VPK_{i}=\sum_{j=1}^{n}h_{j}Y_{j}$, where $h_{j}$ is the jth bit value of a n-bit string and $Y_{j}$ is the jth bit value of the master public key space; $H_{0}\left(ID\right)$ for j=1,2,⋯,n.
- For every $ID_{i}$, i=1 to r, evaluates whether the verification equation $m_{i}P=$ $H_{1}\left(\mu_{i}\right)VPK_{i}+\vartheta_{i}\mu_{i}$ holds. If any of the tuples $\langle m_{i},\mu_{i},\vartheta_{i}\rangle$ does not satisfy the verification equation, the RSU rejects the AVM; otherwise proceeds with verification.
- Extracts the polynomial by picking randomly r−t pairs of $\langle r_{i},Enc\left(k,m_{i}\right)\rangle$ in AVM and a pair $\langle 0,H_{3}\left(t∥r\right)\rangle$ to reconstruct a polynomial f that meets conditions: deg(f)=r−t, $f\left(0\right)=H_{3}\left(t∥r\right)$, and $f\left(r_{i}\right)=Enc\left(k,m_{i}\right)$ for i = 1 to r−t.
  *Verifies whether the remaining pairs of*$\langle r_{i},Enc\left(k,m_{i}\right)\rangle$*in the AVM hold to*$f\left(r_{j}\right)=Enc\left(k,m_{j}\right)$*. If any of the pairs do not hold, rejects the signature; otherwise, the receiver*RSU*accepts and believes the AVM is valid.*

*B. Blockchain-enabled incentive trust model*

The proposed trust model is composed of the following phases: Verification phase, Trust Computation Phase, Incentive-Payment Phase, Consensus phase, and Warning and Removal (Revocation) phase.

**Verification phase:**

The RSU checks the validity of the aggregation message (as mentioned in the last step of the TRS scheme) and then generates the trust value for the sender. Meanwhile, in order to prevent sending false traffic information, the sender $S_{i}$ deposits a certain amount of money on the blockchain. If AVMs are not valid, this deposit is deducted by the RSU from the senders’ accounts on the blockchain at the time of generating incentive transactions for the other sender vehicles whose AVMs are valid. MSG contains all aggregate messages $AVM_{i}$ sent by the senders about the event and is denoted as $MSG=\left(AVM_{1},\ldots ,AVM_{n}\right)$. The incentive will be paid to the sender $S_{i}$ and witness $W_{i}$ by RSU if the message sent by the sender $S_{i}$ is true. Otherwise, $S_{i}$ will lose the deposit for sending false information.

**Trust Computation Phase:**

The indirect trust/recommendation degree is given by the following,

(1) $$IT\left(RSU_{i},S_{i}\right)={\left[\left(\frac{\alpha }{\alpha +\beta }\times \sum_{j=1}^{n}PR\right)+\left(\frac{\beta }{\alpha +\beta }\times \sum_{k=1}^{m}NR\right)\right]}^{\frac{1}{N}}$$

where $IT\left(RSU_{i},S_{i}\right)$ is the recommendation degree of the other senders $S_{j}$ and $S_{k}$ regarding the event message sent by the sender $S_{i}$. *PR* is the number of senders $S_{j}$ who agree with the event message and NR is the number of senders $S_{k}$ who disagree with the event message, α represents the reward factor and *β* represents the penalty factor given based on the total of old trust values of vehicles. *N* represents the number of senders reporting the aggregate message ($AVM_{i}$) about the same event. Then, the global trust of a sender $S_{i}$ is given by

(2) $$T\left(RSU_{i},S_{i}\right)={\left[\prod_{N}{\left(T_{Old}\left(RSU_{i},S_{i}\right).IT\left(RSU_{i},S_{i}\right)\right)}^{\frac{1}{2}}\right]}^{1/N}$$

Moreover, if the global trust $Trust\left(RSU_{i},S_{i}\right)$ of a sender vehicle exceeds a predefined threshold, the vehicle is considered to be trusted. The $RSU_{i}$ adjusts the detection threshold (DetectionTH) when it detects that the trust of the sender has declined. It is then able to quickly detect malicious behavior. As a result, our detection threshold is not a fixed one. It is adaptive based on the behavior of the sender. As trust ranges between 0 and 1, the detection threshold should vary between [0.5, 1]. We store two trust evaluations for every sender vehicle: old trust $T_{Old}\left(RSU_{i},S_{i}\right)$, and new trust $T_{New}\left(RSU_{i},S_{i}\right)$. In every case where the new level of trust is less than the old level, the DetectionTH is raised. When both trust values (old and new) stay the same, the threshold stays the same. Therefore, if the sender vehicle is considered to be honest and cooperative ($T_{New}\left(RSU_{i},S_{i}\right)>T_{Old}\left(RSU_{i},S_{i}\right)$, the lowest detection threshold (0.5) will be applied.

The difference between the new and old trust computations is indicated by $T_{diff}$($T_{diff}=T_{Old}\left(RSU_{i},S_{i}\right)-T_{New}\left(RSU_{i},S_{i}\right)$. The following equation summarizes the detection threshold adaptation in response to vehicle behavior.

(3) $$DetectionTH_{New}=\{\begin{array}{c} \begin{array}{c} DefaultTH+T_{diff}\\ DetectionTH_{old}\\ DefaultTH \end{array} \end{array}\begin{array}{c} ifT_{diff}>0\\ ifT_{diff}=0\\ ifT_{diff}<0 \end{array}$$

**Decision-Making Phase:**

A road condition with the claims $AVM_{A}$ and $AVM_{B}$ where the average trust of the senders $S_{i}$ whose messages claiming the event described in $AVM_{A}$ is greater than the average trust of those senders $S_{i}$ whose messages claiming the event described in $AVM_{B}$ can be indicated by $Confirm\left(AVM_{A},MSG\right)=true$, and $Confirm\left(AVM_{B},MSG\right)=false$. Since $Confirm\left(AVM_{A},MSG\right)=true$, the event described in $AVM_{A}$ is considered to be true. Therefore, $S_{i}$ and $W_{i}$ of $AVM_{A}$ can receive the incentive amount and $S_{i}$ receives his deposit back in the incentive-payment phase. Consequently, the RSU broadcasts an event notification to vehicles within its communication range.

**Incentive-Payment Phase:**

In this phase, the RSU incentivizes the senders and witnesses of $AVM_{A}$ whose signatures and AVM are valid and their trust values $T\left(RSU_{i},S_{i}\right)$ are greater than the detection threshold and $Confirm\left(AVM_{A},MSG\right)=true$. Every sender $S_{i}$ and their witnesses of $AVM_{A}$ can acquire the incentive amount and every $S_{i}$ can acquire the deposit amount back. Every malicious sender $S_{i}$ of $AVM_{B}$ will lose their deposit since $Confirm\left(AVM_{B},MSG\right)=false$. Incentives are not provided to vehicles that do not provide the correct event information. The incentive is added to the participating vehicles’ accounts by RSU and deducted from RSU’s account. The RSU stores the trust value, as well as the incentive, amounts on the blockchain.

**Consensus Phase:**

Our model adopts the PBFT consensus mechanism to obtain a consensus among RSUs. After each interval of window time t, one RSU is randomly selected as the primary (leader) node, and the rest are secondary nodes (validator nodes). The elected primary node generates and transmits a block containing transactions of vehicles’ trust values to all validator nodes (pre-prepare). They next compare the content of the received block to the shared memory pool of transactions to check if the transactions and values in the received block are valid. A prepare message is issued to all other nodes if the comparison is successful (prepare). The primary node broadcasts a commit message to all validator nodes when it receives prepared messages from two-thirds of the validator nodes (commit). When at least two-thirds of their peers send commit messages, validator nodes commit the block and add it to their blockchain ledgers. Therefore, as long as there are more legitimate RSUs than faulty/compromised RSUs (i.e., honest RSUs exceed 3f + 1), a distributed system is still capable of functioning, regardless of the presence of malicious RSUs. 

**Warning and Removal/Revocation Phase:**

Vehicles with low trust values can be punished by RSUs through warnings and removals (revocation). A vehicle whose trust value is less than the warning threshold $Thr_{warn}$ will be added to the warning list. A warned vehicle must actively disseminate reliable information and improve its trust value over time in order to acquire the vehicular network services. If the trust value of the vehicle is less than the removal threshold $Thr_{remove}$, the vehicle will be put on the removal list. This list of vehicles is forwarded to the TA to revoke them from the vehicular network. No services will be provided to these vehicles.

## 5. Security Analysis

In this section, we assess whether the proposed TRS scheme and the blockchain-enabled incentive trust model comply with the security, privacy, and trust requirements of VANETs. 

**Unforgeability**

We consider the security notion that if an adversary can forge more than r−t random $\langle r_{i},Enc\left(k,m_{i}\right)\rangle$ that meets condition deg(f)=r−t, he is capable of generating an EC-ElGamal signature (μ,ϑ) of any message m that satisfies the verification equation $m_{i}P=$ $H_{1}\left(\mu_{i}\right)VPK_{i}+\vartheta_{i}\mu_{i}$. However, assuming the one-way hash functions are hard to break, the probability of success is considered negligible. In addition, it is well known that no effective formula for solving the Lagrange interpolation equation with a degree greater than five exists. As a result, in the proposed TRS scheme, no adversary can forge the EC-ElGamal signature without knowing the private key that satisfies the verification equation. 

**Privacy preservation**

We consider the AVM and RRM messages to demonstrate how our scheme protects the participants’ privacy. By using the threshold ring signature, the sender’s *S* privacy is protected when broadcasting the AVM. The TRS technique requires at least t signers (the sender S included) to sign the AVM while their identities remain anonymous. The AVM consists of the signers’/witnesses’ identities $A=\left\{ID_{r-t+1},ID_{r-t+2},\cdots ,ID_{r}\right\}$ and the non-signers’ identities $\overset{´}{A}=\left\{ID_{1},ID_{2},\cdots ,ID_{r-t}\right\}$. Therefore, a receiver R cannot tell the actual signer because the signers’ group A is hidden in the larger group $A\cup \overset{´}{A}$. The receiver R is sure that t out of r vehicles participated to generate the AVM but cannot identify them. The RRM generated by the witness W is a meaningless random string. Thus, there is no way for a probabilistic polynomial time adversary to link send an RRM to a witness W. Therefore, the proposed TRS scheme protects the participant’s privacy.

**Message authentication and reliability**

The EC-ElGamal signature guarantees the integrity of the AVM. Suppose an adversary wants to tamper with the contents of the message msg in a legitimate way, he must collaborate with t malicious vehicles to receive enough RRMs to produce a deceiving AVM. Moreover, the symmetric encryption technique used in our scheme ensures that the verification equation $m_{i}PH_{1}\left(\mu_{i}\right)VPK_{i}+\vartheta_{i}\mu_{i}$ is specific to a message and cannot be reused to verify other messages originating from the adversary.

**False message attack**

False messages may be broadcast by malicious vehicles in order to mislead or disturb RSUs. The reliability of an AVM is directly related to the number of witnesses who have attested it. The larger the threshold value t, the more the receiver RSU believes the AVM. An adversary may deliberately modify the threshold value t on the AVM to coerce the receiver RSU to accept a deceiving AVM. However, the receiver RSU will detect the malicious activity because the verification equation $m_{i}PH_{1}\left(\mu_{i}\right)VPK_{i}+\vartheta_{i}\mu_{i}$ cannot hold with a modified threshold value t. Hence, the proposed TRS scheme can resist false message attacks. Furthermore, if the sender vehicles have too low trust values the RSU will first warn them, and if they continue to send bogus messages, it will report them to the TA to revoke them from the network. In addition, the sender vehicle that sends bogus event messages will lose the deposited amount.

**Sybil attack**

The proposed TRS scheme leverages the Lagrange polynomial, where t vehicles with different private keys collaborate to generate a threshold ring signature on AVM. Suppose a malicious sender S creates more than r−t fake identities to generate a deceiving AVM. Any receiver RSU will reject the AVM during verification because no probabilistic polynomial time adversary can pick more than r−t random $\langle r_{i},Enc\left(k,m_{i}\right)\rangle$ that meets condition deg(f)=r−t. In the event, that the adversary sender S adjusts r and t and succeeds in generating a WRM with more than r−t random $\langle r_{i},Enc\left(k,m_{i}\right)\rangle$ tuples, any witness W will detect the malicious activity when reconstructing the polynomial f. In addition, for a particular WRM, all r−t tuples in it should be identical. Otherwise, witness W will reconstruct different polynomials $f^{\prime }$ that fail the aggregate message verification stage. In addition, for a specific WRM, a malicious witness W cannot reply to more than one RRM because the verifying equation $m_{i}P=$ $H_{1}\left(\mu_{i}\right)VPK_{i}+\vartheta_{i}\mu_{i}$ is linked to the ID of the witness W. Therefore, adversaries cannot forge identities in our scheme to conduct malicious activities without detection.

**Replay attack**

In the proposed TRS scheme, each AVM contains a message msg and a timestamp, among other contents. If an attacker replays valid AVMs to congest the VANET, a receiver RSU can tell if the AMV is fresh or replayed by checking whether the timestamp on message msg and the current time is a valid time interval. A probabilistic polynomial time adversary must forge a valid EC-ElGamal signature to perform replay attacks. However, the probability of success is considered negligible. 

**Defense against Byzantine RSUs**

As stated in the proposed scheme, an attacker may control a small portion of RSUs. Malicious RSUs may alter or delete data. The PBFT consensus mechanism ensures that the network continues to run normally despite damage to 33% of the RSU nodes. According to PBFT, the block proposer must receive at least 2/3 of the votes from honest secondary RSUs. If the network contains f malicious RSU nodes, and a total of *n* honest RSUs satisfy n ≥ 3f + 1, then the system will be able to defend against malicious data-tampering attacks. Therefore, the proposed system is Byzantine fault-tolerant, which reduces the impact of compromised RSUs. This ensures the security and consistency of the entire network.

**On-off attack**

Using the threshold adaptive control technique, the proposed trust model can detect and exclude attackers who avoid detection by intelligently adapting and varying their behavior in the network.

**Collusion attack**

Malicious vehicles collaborating together will not be able to perform the bogus message attack, since their messages are evaluated by the trust model based on the digital signature, trust score, and the deposited amount.

**Decentralization**

RSUs pay the incentive amounts to the participating senders and their witnesses so our system runs independently without any third party to realize the payment. In addition, trust values are calculated by RSUs based on the aggregate messages received from the senders and are stored in the blockchain ledger in the RSU, ensuring the system’s scalability and reliability.

## 6. Performance Evaluation

**Figure 5:**
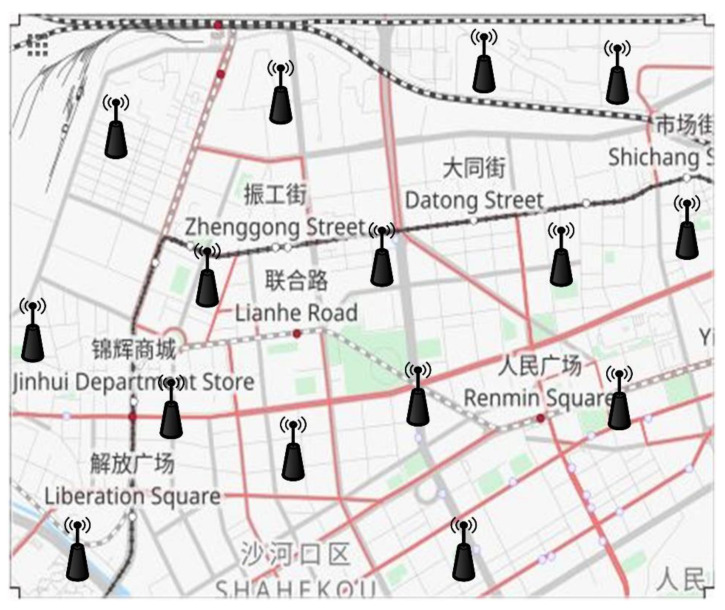
Network Map.

In this section, our proposed system is evaluated through extensive simulations to validate its effectiveness and reliability. We analyze the cryptographic operations involved in the proposed TRS scheme to assess its computation efficiency. To implement our scheme, we utilize the Mbed TLS [39] cryptographic library and the GMP [40] math library with the NIST curves. The simulations are run on a Windows machine using Intel^®^ Core™ i7-3770 CPU @3.40 GHz and 8 GB RAM. We assume the OBUs have the same computing capacity as a modern PC. Hence, the simulation results are practical in a VANET environment. In addition, we analyzed our TRS and trust model by using the simulation platform Veins [41], which is a hybrid framework for running simulations of vehicular networks, OMNet++ works as the network simulator [42], and SUMO works as the road traffic simulator [43]. We import a map from the OpenStreetMap [44] to validate the proposed system as shown in Figure 5. Table 1 contains the configuration parameters used to evaluate the effectiveness of the TRS scheme and trust model.

This section consists of three parts. The first part provides an evaluation of the TRS scheme. The efficiency of trust computation is evaluated in the second part. The third part demonstrates the validity of the consensus method.

*A.*  *Evaluation of the TRS Scheme*

**Figure 6:**
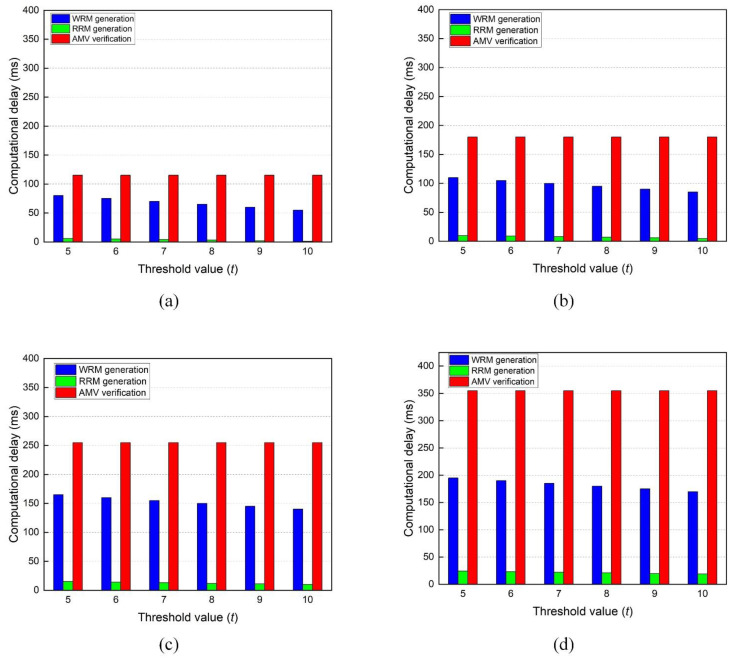
The cryptographic delay during WRM generation, RRM generation, and AVM verification in the proposed TRS scheme. For experiments (**a**–**d**), the ring-size r is fixed at 20, 30, 40, and 50, respectively.

The most cryptographically heavy phases in the proposed TRS scheme are witness-request, request-reply, and aggregate message verification, corresponding to WRM generation, RRM generation, and AVM verification, respectively. We test their computational delay by setting up four experiments (a, b, c, d). We define a fixed ring size r for each experiment and vary the threshold value t. The results are presented in Figure 6a–d.

From Figure 6, the computational delay during WRM generation and RRM generation declines as we increase the threshold value t under a constant ring size r. This owes to the fact that the most expensive computation in our scheme is forging identities, and the number of forged identities is determined as r−t. For example, in Figure 6d, when t = 5, the sender S needs to create 45 fake identities, and when t = 10, she is required to forge 40 identities. Surprisingly, the computation delay of AVM verification is independent of the threshold value and dependent on the ring size. The reason is that all signatures (both actual signatures and forged) are indistinguishable, and the receiver RSU verifies them equally. Therefore, the delay due to the AVM verification is only influenced by ring size regardless of the threshold value. The computation delay of request-reply is significantly tiny relative to the other two phases. This is advantageous because witnesses can instantly send RRMs to the sender S, which enhances the efficiency of AVM generation. In summary, according to Figure 6a–d, the proposed TRS scheme has an acceptable computational delay in VANETs.

**Anonymity**

Furthermore, the ring size and the threshold value impact the anonymity of the ring members. The anonymity of our scheme has two perspectives: the probability an attacker can correctly identify the actual signer and the probability an attacker can correctly extract the number of witnesses from an AVM. Essentially the success probability of these perspectives is the ratio of the threshold value t to the ring size r. When the difference r−t is slight, the anonymity level is low, and when the difference r−t is significant, the anonymity level is high. Witnesses could be willing to endorse a WRM since it is a cryptographically cheap exercise. However, anonymity needs to be guaranteed. Therefore, choosing a threshold value t and a ring size r is a dilemma that every sender S should balance when generating a WRM.

We evaluate the cryptographic computation cost of the proposed TRS scheme by considering the cost of generating and verifying a signed message. In addition, we compare our scheme with other threshold ring-signature solutions by Liu et al. [45] and Mei et al. [46]. The comparisons are presented in Table 2.

The analysis considers ten vehicles that have experienced a traffic event on a particular road and wish to alert other vehicles. In Liu et al. [45], a sender generates a message individually using 127.57 ms, while a receiver requires 255.84 ms to aggregate all messages from the ten vehicles and verify them. The total computation cost in the scheme [45] is 383.41 ms, which is inefficient due to computationally heavy bilinear pairings. Similarly, every sender in Mei et al. [46] scheme generates her message individually within 17.2 ms, whereas a receiver needs 20.4 ms to verify a single message and 204 ms to verify ten messages. Generation and verification in the scheme [46] incur a total cost of 221.2 ms, which is acceptable in VANETs. However, verifying messages one by one is inefficient as the number of messages grows.

In the proposed scheme, the cost of generating a message has two steps. First, the sender spends 42.4 ms composing a witness-request message, and then a witness needs 1.6 ms to respond with a reply-request message. Hence, the sum cost of generating an aggregate message in our scheme is 44 ms. We anticipate the witnesses to respond in parallel, and time will not be wasted waiting for replies. On the other hand, a receiver in our scheme requires 126.1 ms to verify an aggregate message, making the total cost of generation and verification 170.1 ms. We attribute this efficiency to our scheme’s combined public key technique and EC-ElGamal signature.

**Availability**

**Figure 7:**
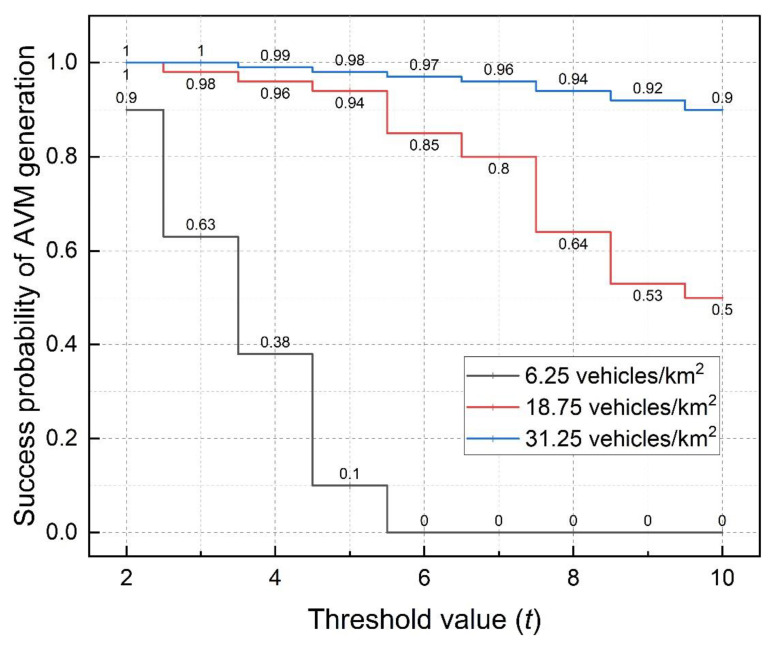
The success probability of an AVM generation.

Availability of the proposed TRS scheme is synonymous with the success rate of producing an AVM. That is, the probability in which a sender S generates an AVM successfully upon broadcasting a WRM and receiving t RRMs from different vehicles. We compute the success probability of AVM generation, which is our scheme’s availability, by dividing the total number of received RRMs by the threshold value t. We simulate by varying the threshold values and vehicle densities (vehicles/km^2^) and show the results in Figure 7. We fix the ring-size r at 20 since it has a negligible effect on availability.

From Figure 7, there is a high probability for the sender S to generate an AVM when the vehicle density is high and the threshold value is low. This is due to the ease of finding witnesses. Meanwhile, witnesses are motivated to endorse AVMs when anonymity is assured. Therefore, we recommend deploying the proposed TRS scheme in dense networks such as cities.

The cryptographic and non-cryptographic delays also affect the availability of our scheme. The former refers to the processing delay during signature generation and verification, and the latter corresponds to the network performance, mainly the transmission delay. We evaluate the non-cryptographic delay by measuring the time spent in round communication between the sender and the witnesses. In other words, the time difference between broadcasting a WRM and generating an AVM. 

**Figure 8:**
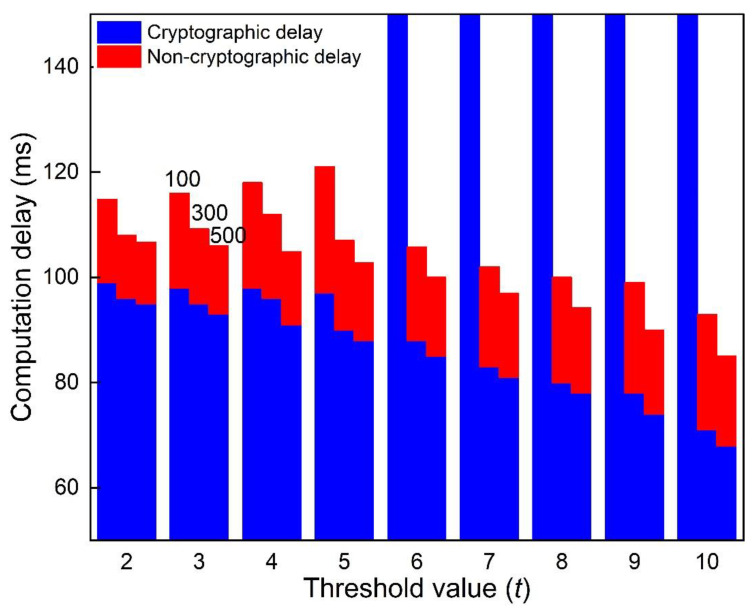
A comparison of cryptographic delay and non-cryptographic delay at various threshold values and number of vehicles (100, 300, 500).

We exclude AVM verification from this discussion because it depends on the receiver’s processor speed regardless of the network condition. Fixing the ring-size r at 20, we simulate and present the results in Figure 8.

Figure 8 depicts that the cryptographic delay is significantly greater than the non-cryptographic delay. The reason is that the transmission delay is very low, enabling instant responses of witnesses. It is worth noticing that the number of vehicles influences our scheme’s availability. For instance, the AVM generation failed at 100 vehicles and a threshold value of 6. This owes to the time-out as the sender S waits for t RRMs. When the vehicle density is high, the sender will likely find witnesses within the WRM’s time-to-live. Therefore, we strongly discourage vehicles from generating the AVMs in sparse networks and high threshold values.

*B.*  *Evaluation of blockchain-enabled incentive trust model*

We used several parameters to evaluate the efficiency of the trust model: True Positive Rate (*TPR*), True Negative Rate (*TNR*), trust metric, and the participation rate of vehicles. The performance of our trust model is compared to BayesTrust [47] model.

- *TPR*: the proportion of malicious vehicles that are classified as untrustworthy. It is presented in the following equation:
  (4) $$TPR=\frac{TP}{TP+FN}$$
  where a True Positive (*TP*) is the number of vehicles that are correctly classified as malicious. In addition, a False Negative (*FN*) is the number of vehicles that have been incorrectly identified as legitimate.
- *TNR:* the proportion of legitimate vehicles that are classified as trustworthy. It is shown in the following equation:
  (5) $$TNR=\frac{TN}{TN+FP}$$
  where True Negatives (*TN*) are vehicles that have been correctly identified as legitimate. False Positives (*FPs*) are the vehicles that were incorrectly identified as malicious.

**TNR and TPR of Trust Model under Collusion Attacks**

**Figure 9:**
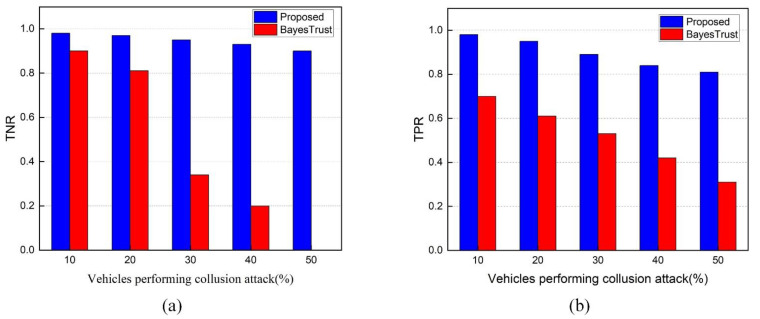
Experiments (**a**,**b**) represent TNR and TPR under collusion attack.

Figure 9a,b illustrate BayesTrust’s and our scheme’s *TNR* and *TPR* metrics under different percentages of collusion vehicles. Since BayesTrust has no mechanism to defend against collusion attacks, the two metrics of BayesTrust become worse as the proportion of collusion vehicles increases. The *TNR* and *TPR* of BayesTrust are 0 and 0.31 when 50% of the vehicles are collusion vehicles, which indicates that all legitimate vehicles are considered untrustworthy, and part of the colluding vehicles are considered trustworthy. 

When compared to BayesTrust, our scheme achieves higher *TNR* and *TPR*. When 50% of the nodes are collusion vehicles, the *TNR* and *TPR* of our scheme are 0.9 and 0.81, respectively, indicating that only a small percentage of collusion vehicles are incorrectly classified as trustworthy. It shows that our scheme is far more resistant to collusion attacks in VANET than BayesTrust. Our scheme is able to defend against collusion attacks because it is based on (1) a trust metric that combines the old and new trust values and (2) the deposited amount by the sender.

In general, the chances of successfully recognizing the event state diminish as the number of malicious vehicles grows. A false event spreads over the network faster if there are more attackers, negatively impacting the other RSU decisions. The high ability of our scheme to detect malicious vehicles suggests that it can filter out the false information that spreads across the network and hence is highly resistant to attacks based on false information.

**TNR and TPR of Trust Model under On-Off Attacks**

**Figure 10:**
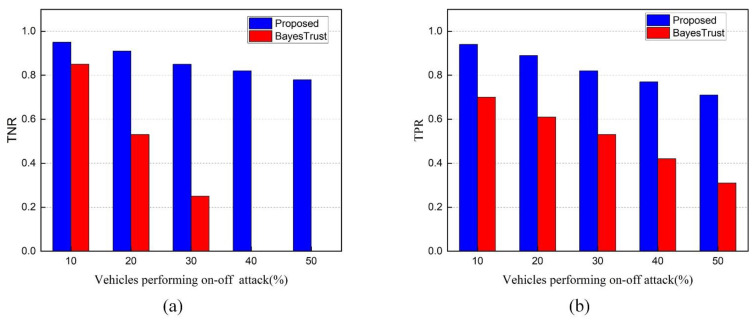
Experiments (**a**,**b**) represent TNR and TPR under On-off attack.

In Figure 10a,b, on-off attackers distribute false information throughout the network to deceive RSUs and they behave intelligently by changing their behavior from honest to malicious and vice versa. The introduction of an on-off attack had a significant impact on TNR and TPR, as seen in Figure 10a,b. Our trust model is effective at detecting attackers who adopt the on-off attack pattern. The reason for this is that our trust model is based on a trust metric that combines the old trust value and the new trust value and employs the adaptive-detection threshold. Additionally, the overall performance significantly decreased by increasing the on-off attack pattern in the network. In terms of *TNR* and *TPR*, our trust model is more accurate than BayesTrust. The *TNR* and *TPR* of the proposed trust model are around 0.78 and 0.71 where 50% of vehicles are malicious, whereas the BayesTrust is about 0 and 0.31.

The figures illustrate how our proposed scheme detects malicious vehicles within the network. With the adaptive-detection threshold, our solution shows a higher detection ratio over BayesTrust even if there is a large number of intelligent attackers.

**Trust metric**

The trust metric is an evaluation metric that portrays how well the trust model is able to detect and classify legitimate and malicious messages. 

**Figure 11:**
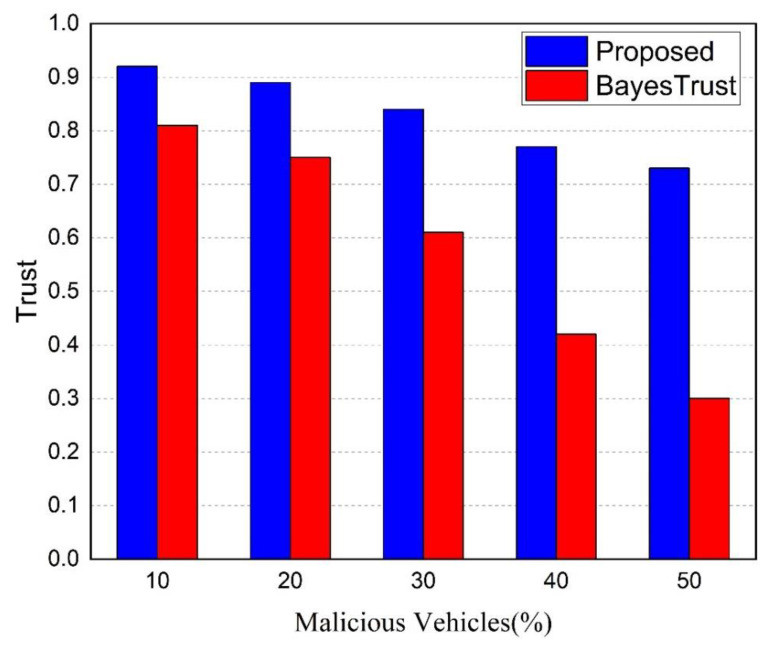
Trust metric.

Figure 11 illustrates the efficiency of the proposed trust model and BayesTrust model for identifying and classifying malicious content from a trust perspective in the presence of malicious vehicles. It shows that trust in the network declines when malicious vehicles generate false messages on the network. The reason for this is that higher malicious vehicles limit RSUs’ ability to detect true events when malicious content is spread in the network. 

Compared to BayesTrust, the proposed trust model exhibits a higher trust value, indicating that it is capable of providing accurate identification and classification of true events, even in the presence of malicious vehicles. This is due to two factors: (1) the trust check of aggregate messages received from multiple senders and (2) each sender’s deposit amount allows the RSU to distinguish between legitimate vehicles and attackers. When there are 50% malicious vehicles in the network, the proposed trust model achieves 73% trust, while BayesTrust achieves a level below 50%.

**The participation rate of vehicles**

Participants’ willingness to participate in the event-validation process is indicated by their participation rate. Incentives will increase the willingness of senders and witnesses to participate in event validation while preventing selfish behavior. The participation rate is calculated according to the following equation,

(6) $$ParticipationRate=\frac{N_{ini}}{N_{tot}}$$

where $N_{tot}$ represents the total number of vehicles present at the event location, and $N_{ini}$ represents the vehicles that agreed with the sender.

**Figure 12:**
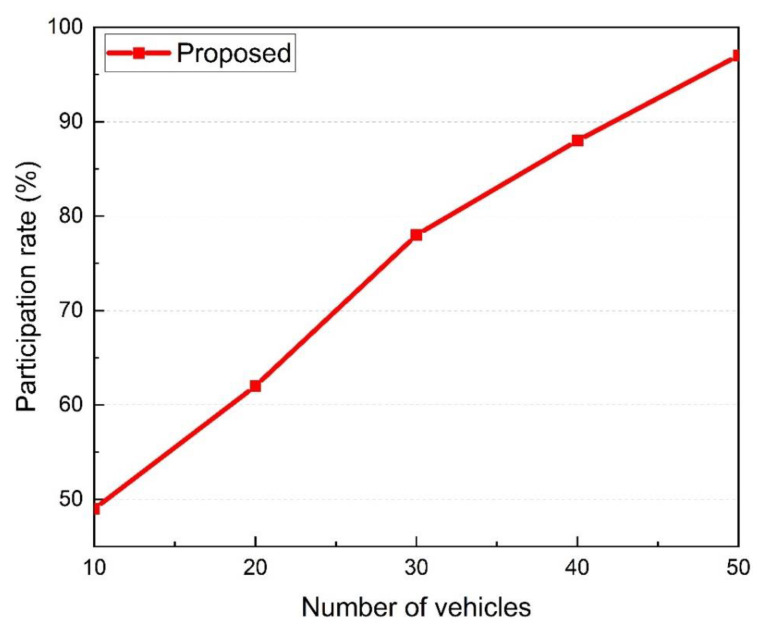
The participation rate.

The proposed approach incorporates incentives to increase the rate of vehicle participation in event validation. Only monetary incentives are included in our scheme. The proposed system was found to be effective at increasing vehicle willingness to confirm event information and, as a result, increasing participation rates. As shown in Figure 12, the participation rate approaches 100% when 50 vehicles are present. This indicates that nearly every vehicle is involved in signing events.

*C.*  *Validity of the Consensus Mechanism*

**Figure 13:**
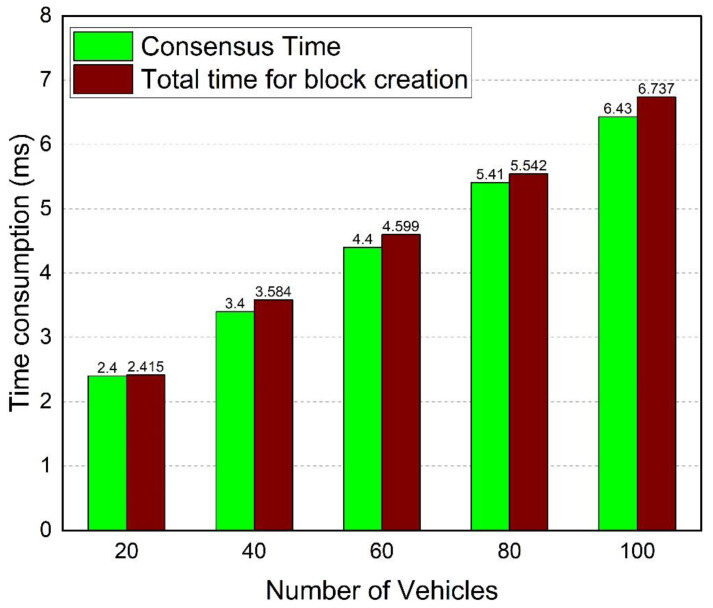
The time taken for block creation and consensus.

We evaluate the time required to create a block and reach a consensus in this part. The total time necessary to construct each block and the time taken to achieve consensus amongst RSUs using PBFT are shown in Figure 13. For a network of 100 vehicles and a maximum of 15 RSUs, it takes 6.737 milliseconds to create a new block. As a result, blocks are generated in a short period of time. Moreover, a new block of an average size of 1.9 KB is created every 100 s.

## 7. Discussion

In Liu et al. [45], the sender requires 127.57 ms to generate a signed message (i.e., three scalar multiplications and a bilinear pairing operation). At the same time, the receiver needs 255.84 ms to aggregate and verify the ten signed messages in a batch, which entails two bilinear pairings and two scalar multiplications. The total computation cost in the scheme [45] is 383.41 ms. The scheme has a shorter presentation of group elements and provides an approximate secure level. However, it is inefficient due to computationally heavy asymmetric bilinear pairings.

According to Mei et al. [46] scheme, the sender vehicle collaborates with random ring members to generate a ring signature. In addition, its ring size is non-deterministic. For instance, when the ring members reach 20, a sender in Mei et al. generates her message within 17.2 ms, which involves two scalar multiplications. To verify an individual signature, a receiver requires 20.4 ms, which entails two scalar additions and two-point additions. Therefore, to verify ten messages from ten vehicles, a receiver needs 204 ms. Generation and verification in the scheme [46] incur a total cost of 221.2 ms, which is acceptable in VANETs. However, verifying messages one by one is inefficient as the number of messages grows.

The scheme we propose is based on Lagrange interpolation, while schemes [45,46] are based on bilinear pairing and ECC, respectively. In our scheme, the cost of generating a message has two steps. First, the sender spends 42.4 ms composing a witness-request message, and then a witness needs 1.6 ms to respond with a reply-request message. Hence, the sum cost of generating an aggregate message in our scheme is 44 ms. We anticipate the witnesses to respond in parallel, and time will not be wasted waiting for replies. On the other hand, a receiver in our scheme requires 126.1 ms to verify an aggregate message, making the total cost of generation and verification 170.1 ms. We attribute this efficiency to our scheme’s combined public key technique and EC-ElGamal signature. 

The percentage improvement of our scheme over Liu et al. [45] on signature generation and verification is 65.51% and 50.71%, respectively, and 55.63% advantage on the total cost. Compared with Mei et al. [46], the proposed scheme depicts a 60.9% disadvantage when generating a signature but boasts 38.19% efficiency during verification and 23.10% efficiency on the total cost.

Additionally, as discussed in the security analysis section, the proposed TRS scheme can guarantee anonymity, reliability, trust, and efficiency in a VANET scenario. 

BayesTrust model [47] is primarily driven from the webpage-ranking algorithms such as PageRank. Due to random topology and dynamic connections, the VANET does not have an explicit link structure; nonetheless, social trust relationships between vehicles do exist, and an implicit web of trust can be derived. BayesTrust and VehicleRank are the two algorithms that make up this system. BayesTrust adopts methods from Bayesian statistics and is used to calculate local trust values. Through VehicleRank, which builds an implicit network of trust and applies link analysis algorithms, global trust values are calculated. The receiver vehicles in this system use the global trust values calculated by the above two algorithms to decide whether to trust the message sent by the neighboring vehicle or not. 

Our trust model verifies and evaluates the received aggregate messages sent by vehicles to the RSU. It calculates the trust values of these vehicles by first calculating the indirect trust value and then calculating the global trust value of every sender vehicle. Then, the calculated trust value (global trust value) is compared with the adaptive-detection threshold to decide whether the sender vehicle is malicious or not. 

The *TPR* and *TNR* metrics are used to determine the efficiency of the trust model. The higher the *TPR* and *TNR* values, the higher the ability of the trust model to identify malicious and legitimate vehicles. When there are 50% malicious vehicles in the network, the proposed solution achieves *TNR*(90%) and *TPR*(50%) percentages better than BayesTrust in the case of collusion attacks, *TNR*(78%) and *TPR*(40%) better than BayesTrust in the case of on-off attacks and higher trust level (43%) than BayesTrust in the network. The incentive mechanism achieves a 100% vehicle participation rate. We attribute the better performance of our trust model to the TRS scheme, which ensures the authenticity and reliability of shared messages between sender vehicles and the RSUs and the additional trust checks made at the RSUs level. Moreover, our trust model has a tracing and revocation mechanism to trace and revoke identified malicious vehicles, and these abilities are not provided by the BayesTrust model. Furthermore, the identity privacy of vehicles is protected by our trust model, while this VANET security requirement is not satisfied by the BayesTrust model. 

Due to the inclusion of the incentive mechanism, which is not present in the BayesTrust model, our proposed trust model was proven to be effective at improving vehicle willingness to validate event information and, as a result, increasing participation rates. 

By adopting the PBFT consensus mechanism, our proposed system constructs each block and achieves consensus to guarantee that trust values are distributed and synchronized amongst RSUs in a short time span measured in milliseconds. 

However, the key management issue is a limitation in our scheme due to numerous new public keys. 

### Continuous Delivery and Deployment

Every software project involves some degree of change. Changing the source code is common when resolving bugs or introducing new features. Consequently, continuous delivery and continuous deployment are crucial factors in determining the success and efficiency of software.

A continuous delivery strategy automates the process of delivering changes made by application developers to the repository or container registry, and it shows how the changes are automatically tested for errors. Therefore, any modifications to the proposed solution software/architecture will automatically undergo bug testing before being pushed to a repository, such as GitHub or a container registry. Finally, the car manufacturers can download the new version of the software application and install it on hardware devices, such as a vehicle’s OBU or RSU. The continuous delivery process ensures minimal effort is required to deploy new changes [48].

Continuous deployment means deploying software changes to the production environment automatically as soon as they are ready, without human intervention [49]. Therefore, any changes made to the proposed solution software/architecture are automatically released from the repository to production, where hardware manufacturers can use them. By automating manual processes, it reduces the burden on operations teams. This practice automates the next step in a continuous delivery process, adding to the benefits of continuous delivery.

Therefore, in case of changes, the adopted continuous delivery/continuous deployment practices can help to deliver and deploy software releases quickly and safely.

Concerning software delivery and deployment, additionally, we leverage Hyperledger Fabric, a blockchain automation framework. The choice of the Hyperledger platform is inspired by its ability to abstract complexity. In addition, the car manufacturers can specify required hardware, latency, throughput, availability, and threat models. However, the main limitation is the lack of support for heterogeneous blockchain networks, such as multi-channel topology and multi-technology. 

## 8. Conclusions

In this paper, we presented blockchain-enabled trust management combined with a TRS scheme. The TRS scheme is efficient in the VANET environment that is not fully trusted because it maintains the high authenticity and reliability of aggregate messages without revealing users’ privacy. Our method requires numerous senders and their witnesses to provide traffic data to the nearest RSU. Each RSU determines the trustworthiness of the aggregate messages sent by the senders regarding a particular event. Using the proposed approach, all vehicles would be able to send anonymous messages and distributed RSUs would be able to receive traffic information, evaluate and update vehicle trust values, as well as offer incentive amounts for participating vehicles. With the incentive mechanism, the RSU provides rewards to senders and witness vehicles to encourage them to overcome their selfish behavior and deliver true traffic data. This strategy increases vehicle participation in the event-validation process. It ensures that participating senders and their witnesses either receive payment (by delivering real traffic information) or lose their deposit (by providing false traffic information). The performance comparison of the total cryptographic computation cost shows that our scheme is 55.63% more efficient than Liu et al.’s scheme and 23.10% more efficient than Mei et al.’s scheme. Therefore, we endorse our proposal to be applied in VANETs. In addition, the trust metric shows that our trust model is 0.43% more efficient than the BayesTrust model when there are 50% malicious vehicles in the network. The evaluation of our TRS scheme shows that it can efficiently work compared with the related schemes. Furthermore, the proposed trust model achieves efficient, secure, and robust trust evaluation, identifies malicious vehicles, resists various kinds of attacks, and revokes malicious vehicles from the vehicular network. The security analysis and performance evaluation show that our method is effective and feasible for vehicular networks.

In the future, we intend to address the key management issue and consider constructing an efficient threshold ring signature that is provably secure in the post-quantum setting.

## Figures and Tables

**Table 1:** Simulation Details.

Parameters	Values
Simulation period	1000 s
Size of area	4 km × 4 km
Number of vehicles	100/300/500
Number of malicious vehicles (%)	10, 20, 30, 40, 50
Vehicle speed	40–70 km/h
Number of RSUs	15
Network Protocol	WAVE
MAC Protocol	IEEE 802.11p
Transmission range	300 m
AddBlockTimer	100 s
InitialTrust	0.3
Trust threshold (DefaultTH)	0.5

**Table 2:** Comparison of cryptographic computation cost (ms).

Scheme	Generation	Verification	Total
Liu et al. [45]	127.57	255.84	383.41
Mei et al. [46]	17.2	20.4 × 10 = 204	221.2
Proposed	Sender: 42.4	126.1	170.1
	Witness: 1.6		

## Data Availability

Not applicable.
